# Four-factor nomogram for early-onset sepsis in preterm neonates: Development and internal validation of a stewardship tool

**DOI:** 10.1371/journal.pone.0334342

**Published:** 2025-10-09

**Authors:** Li Guo, Zhiyang Zhang, Chunhui Zhao, Cuncun Shen, Haotian Zhao, Huifen Chen

**Affiliations:** 1 Department of Neonatal, The Fourth Hospital of Shijiazhuang, Shijiazhuang, China; 2 Department of Intensive Care Unit, Hebei General Hospital, Shijiazhuang, China; 3 Department of Ultrasound, Hebei General Hospital, Shijiazhuang, China; Saitama Medical Center: Saitama Ika Daigaku Sogo Iryo Center, JAPAN

## Abstract

**Background:**

Early-onset sepsis (EOS) remains a leading cause of mortality and neurodevelopmental injury in preterm infants, yet widely used tools (e.g., Kaiser EOS Calculator) are not designed for <35–37 weeks’ gestation.

**Objective:**

To develop and internally validate a concise, clinically interpretable nomogram for EOS risk stratification in preterm neonates and to evaluate its potential for antibiotic stewardship.

**Methods:**

We performed a single-center retrospective cohort study (July 2023–June 2024) including 1,059 preterm infants admitted within 72 h of birth, randomly split 7:3 into training (n = 742) and validation (n = 317). Forty-five maternal and neonatal candidates were screened (univariable tests, LASSO), followed by multivariable logistic regression to build the final model and nomogram. Discrimination (AUC), calibration (Brier score, calibration curve, Hosmer–Lemeshow), and decision-curve analysis (DCA) were assessed; two biologically plausible interactions were prespecified.

**Results:**

Four routinely available variables—gestational age, birth weight, umbilical cord abnormality, and mechanical ventilation within 72 h—composed the final model. In the validation cohort, AUC was 0.818 (95% CI, 0.767–0.868), Brier score 0.158, and Hosmer–Lemeshow *P* = 0.71; DCA showed net benefit across 5–65% risk thresholds. Using a ≥ 0.70 treatment threshold, the model identified 88% of EOS cases while recommending antibiotics for ~10% of infants. A culture-proven-only sensitivity analysis yielded comparable discrimination (AUC 0.819) with a Brier score 0.041.

**Conclusions:**

A four-factor nomogram using EMR-available variables accurately stratifies EOS risk in preterm infants and may support risk-based antibiotic decisions while limiting overtreatment. Prospective multicenter external validation is warranted to confirm generalizability and guide implementation.

## Introduction

Early-onset sepsis (EOS) remains a major cause of death and neurodevelopmental injury in preterm infants (<37 weeks), whose mortality is 3–5 times higher than in term neonates and is further compounded by early empirical antibiotic use [1–3]. Although empiric therapy broadens initial coverage, it disrupts the microbiome, drives resistance, and may have downstream metabolic harms [4]. To curb these effects, AAP and NICE advocate risk-based initiation; however, widely used tools such as the Kaiser EOS Calculator (designed for ≥35 weeks) do not address key vulnerabilities of very preterm infants—immune immaturity, ventilator dependence, and perinatal hypoxia-ischaemia [5,6]. We therefore developed and internally validated a four-factor nomogram tailored to preterm infants (<37 weeks, including <35 weeks), using routinely available perinatal variables, and evaluated its discrimination, calibration, and decision-curve utility to inform antibiotic stewardship.

While machine learning improves late-onset sepsis prediction, its EOS use remains limited and lacks mechanistic clarity [7]. Many models rely on high-frequency data or complex inputs, hindering clinical use and lacking biological transparency [8]. Key risk factors—such as umbilical cord abnormalities, maternal metabolic disorders (e.g., hyperglycemia, insulin resistance), and ventilator-induced lung injury (VILI)—disrupt immune homeostasis but are often omitted in EOS models [9,10].

This study aimed to develop and internally validate a concise, interpretable EOS risk model using data from preterm infants admitted to a tertiary neonatal hospital (2023–2024). We further assessed its value for NICU stratified management and examined biological interactions to inform personalized antibiotic decisions.

## Materials and methods

### Study design and participants

**Fig 1:**
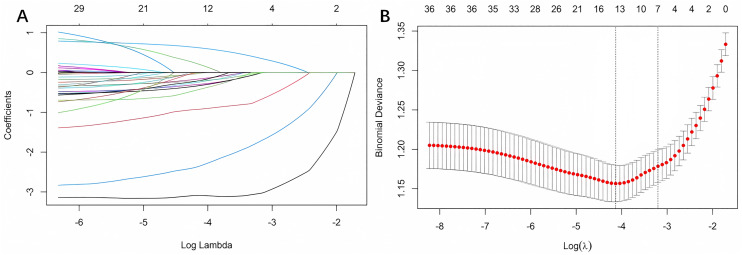
Variable selection process using LASSO regression. (A) Coefficient trajectories of 36 candidate variables across log(λ) values. As λ increases, uninformative variables shrink toward zero. (B) Tenfold cross-validation curve identifying the optimal penalty (λ_min) that minimizes binomial deviance. A total of seven predictors were retained for multivariable modeling based on this selection.

This single-center retrospective cohort study was conducted in the Neonatal Department of the Fourth Hospital of Shijiazhuang (Hebei, China) from July 2023 to June 2024. Ethics approval was obtained (Approval No. 20230134), and written informed consent was obtained from parents or legal guardians and documented in the medical record. All data were de-identified prior to analysis in accordance with institutional policy.. Based on CDC and AAP criteria [5,11], we included live-born preterm infants (GA < 37 weeks) admitted within 72 hours and with complete maternal and neonatal data (<20% missing). Exclusion criteria were major malformations, palliative care, early death (≤72 h), incomplete culture/treatment data, or duplicated records. We excluded infants who died within 72 h to minimize outcome ascertainment bias and because early treatment responses could not be reliably observed. A total of 1,059 infants were enrolled and randomly split (7:3) into training (n = 742) and validation (n = 317) sets using a fixed seed (set.seed = 3464) (Fig 1). Missing-data handling is detailed in Variable Selection and Missing Data Handling; see S1 Table.

### Definition of outcomes

EOS was defined as either: (1) positive culture of pathogenic bacteria from blood or other sterile fluids within 72 hours of birth, or (2) negative culture with typical infection signs (e.g., respiratory distress, temperature instability, reduced consciousness) plus at least one elevated inflammatory marker (CRP ≥ 10 mg/L, PCT ≥ 2 ng/mL, or IL-6 ≥ 50 pg/mL). Clinical improvement after ≥5 days of antibiotics was required. In cases of death occurring during the treatment period, these infants were excluded from the clinical improvement criteria, but their EOS diagnosis was retained if they met the culture or clinical criteria. The definition was based on AAP and CDC guidelines for neonatal infection and GBS screening [5,11].

### Variable selection and missing data handling

We extracted 45 candidate variables, encompassing maternal demographics, antenatal complications, perinatal events, delivery/resuscitation details, early interventions (e.g., corticosteroids, surfactant, mechanical ventilation within 72 h), and initial lab results. To ensure the integrity and accuracy of the analysis, missing data were handled using multiple imputation with the MICE package in R. Continuous variables were imputed using linear regression, and categorical variables with logistic regression. Variables with missingness ≥5% were excluded. Five imputed datasets were generated, and sensitivity analysis was conducted to evaluate the model’s performance across these datasets. The performance metrics (AUC, accuracy, sensitivity, specificity) exhibited negligible variation, indicating high consistency. Given the minimal differences observed, Dataset 4 (denoted with an asterisk) was randomly selected for further analysis (S1 Table). Ultimately, 36 variables with complete data were included. To ensure consistency in binary definitions (e.g., maternal occupation, delivery mode, cord abnormalities), standard criteria are detailed in S2 Table. Nine variables were excluded due to high missingness, ambiguity, or confounding (see S3 Table).

### Statistical analysis

Baseline comparisons were conducted using chi-square/Fisher’s exact tests (categorical) and t-test/Wilcoxon tests (continuous, α = 0.05, two-sided). Missing data handling was performed through multiple imputation, generating five imputed datasets. To ensure the robustness of the model, sensitivity analysis was conducted across these datasets. Variable selection was initially performed with univariate logistic regression (*P* < 0.05), followed by LASSO (glmnet, α = 1, 20-fold CV, λ_min). Overlapping variables were then entered into a multivariate logistic regression model with stepwise selection (min AIC). A nomogram was constructed using the rms package [12]. Model discrimination was assessed using AUC (95% CI), sensitivity, specificity, and the Youden index. Calibration used 1,000 bootstrap resamples, Brier score, intercept/slope, HL test, and E/O ratio. DCA over a 0.05–0.65 threshold range (rmda) evaluated clinical utility. EOS risk was stratified as low (<0.30), moderate (0.30–0.70), and high (>0.70), with chi-square and trend tests used to compare groups. Two interaction terms (cord × gestational age; ventilation × weight) were pre-specified and visualized (interactions). Analyses used R 4.3.2 with glmnet, rms, pROC, ResourceSelection, rmda, interactions, and mice.

### Sensitivity analyses

To benchmark the composite outcome against culture-proven incidence, we conducted a culture-proven–only analysis in the validation cohort. Predicted probabilities were generated without refitting from the model trained in the development data, while infants without or with indeterminate culture results were excluded a priori. Performance metrics included AUC (DeLong 95% CI), Brier score, Hosmer–Lemeshow statistics, a calibration curve, and decision-curve analysis across thresholds 0.05–0.65.

### Sample-size considerations and EPV

**Sample-size considerations**: We did not perform an a priori sample-size calculation tailored for multivariable prediction (anticipated incidence, number of candidate predictors, target shrinkage). To mitigate overfitting, we used LASSO penalization and internal validation (pre-specified split with fixed seed and bootstrap), and we provide full model specification and code.

**Candidate-level EPV**: We initially screened 45 candidate predictors; with 285 EOS events in the training set, the candidate-level EPV ≈ 6.3. We therefore relied on penalization and internal validation rather than post-hoc EPV to support model adequacy.

## Results

### Study population

A total of 1,059 preterm infants were enrolled (Table 1) and randomly assigned to training (n = 742) and validation (n = 317) sets. EOS incidence was comparable between the two sets (38.4% vs. 38.5%), confirming balanced outcome distribution.

**Table 1:** Baseline characteristics of preterm infants in the training and validation cohorts, stratified by early-onset sepsis (EOS) status.

		Training cohort				Validation cohort		
	All infants	Non-sepsis	Sepsis	*P*	All infants	Non-sepsis	Sepsis	*P*
	N = 742	N = 457	N = 285		N = 317	N = 195	N = 122	
Maternal demographics								
Maternal age (years)	31.0(28.0,34.0)	31.0(28.0,34.0)	31.0(28.0,34.0)	0.657	31.6 ± 4.3	31.6 ± 4.3	31.6 ± 4.2	0.940
Maternal occupation				0.844				0.668
No	421(56.7)	258(56.5)	163(57.2)		198(62.5)	120(61.5)	78(63.9)	
Yes	321(43.3)	199(43.5)	122(42.8)		119(37.5)	75(38.5)	44(36.1)	
Maternal smoking				**0.999**				**0.652**
No	734(98.9)	452(98.9)	282(98.9)		312(98.4)	191(97.9)	121(99.2)	
Yes	8(1.1)	5(1.1)	3(1.1)		5(1.6)	4(2.1)	1(0.8)	
Gravidity(n)	1(1,2)	1(1,2)	1(1, 2)	0.288	2(1,2)	2(1,2)	1(1,2)	0.384
Parity(n)	2(1,2)	2(1,2)	2(1,2)	0.333	2(1,2)	2(1,2)	2(1,2)	0.278
Mode of conception				0.248				0.785
No	626(84.4)	380(83.2)	246(86.3)		280(88.3)	173(88.7)	107(87.7)	
Yes	116(15.6)	77(16.8)	39(13.7)		37(11.7)	22(11.3)	15(12.3)	
Maternal complications								
Hypertensive disorders in pregnancy				0.220				0.582
No	537(72.4)	338(74.0)	199(69.8)		223(70.3)	135(69.2)	88(72.1)	
Yes	205(27.6)	199(26.0)	86(30.2)		94(27.9)	60(30.8)	34(27.9)	
Gestational diabetes mellitus				0.586				0.650
No	541(72.9)	330(72.2)	211(74.0)		224(70.7)	136(69.7)	88(72.1)	
Yes	201(27.1)	127(27.8)	74(26.0)		93(29.3)	59(30.3)	34(27.9)	
Intrapartum fever				0.130				**0.679**
No	724(97.6)	449(98.2)	275(96.5)		311(98.1)	192(98.5)	119(97.5)	
Yes	18(2.4)	8(1.8)	10(3.5)		6(1.9)	3(1.5)	3(2.5)	
Premature rupture of membranes				0.954				0.451
No	525(70.8)	323(70.7)	202(70.9)		231(72.9)	145(74.4)	86(70.5)	
Yes	217(29.2)	134(29.3)	83(29.1)		86(27.1)	50(25.6)	36(29.5)	
Placenta previa				0.622				**0.746**
No	718(96.8)	441(96.5)	273(95.8)		307(96.8)	188(96.4)	119(97.5)	
Yes	28(3.8)	16(3.5)	12(4.2)		10(3.2)	7(3.6)	3(2.5)	
Placental abruption				0.739				0.447
No	720(97.2)	443(96.9)	275(96.5)		307(96.8)	190(97.4)	117(95.9)	
Yes	24(3.2)	14(3.1)	10(3.5)		10(3.2)	5(2.6)	5(4.1)	
Abnormal placental pathology				0.466				0.694
No	611(82.3)	380(83.2)	231(81.1)		267(84.2)	163(83.6)	104(85.2)	
Yes	131(17.7)	77(16.8)	54(18.9)		50(15.8)	32(16.4)	18(14.8)	
Perinatal characteristics								
Singleton pregnancy				0.068				0.901
No	507(68.3)	301(65.9)	206(72.3)		230(72.6)	141(72.3)	89(73.0)	
Yes	235(31.7)	156(34.1)	79(27.7)		87(27.4)	54(27.7)	33(27.0)	
Neonatal sex				0.064				0.350
Female	403(54.3)	236(51.6)	167(58.6)		169(53.3)	108(55.4)	61(50.0)	
Male	339(45.7)	221(48.4)	118(41.4)		148(46.7)	87(44.6)	61(50.0)	
Gestational age (weeks)	35(34,36)	35(34,36)	34(32,36)	<0.001	35(34,36)	35(34,36)	34(32,35)	<0.001
Additional gestational days	3 (1,5)	3(1,5)	3(1,5)	0.205	3(1,5)	3(1,5)	3(1,4)	0.325
Birth weight (g)	2305.0 ± 519.6	2471.9 ± 414.8	2103.6 ± 594.2	<0.001	2326.9 ± 540.5	2501.6 ± 427.1	2028.7 ± 583.9	<0.001
Birth length (cm)	47(45,49)	48(45,51)	46(43,49)	<0.001	46(45,49)	47(45,49)	44(43,45)	<0.001
Head circumference (cm)	33(32,33)	33(33,33)	32(31,33)	<0.001	32(31,33)	33(32,33)	31(30,32)	<0.001
Birth asphyxia				0.684				0.071
No	558(75.2)	346(75.7)	212(74.4)		236(74.4)	152(77.9)	84(68.9)	
Yes	184(24.8)	111(24.3)	73(25.6)		81(25.6)	43(22.1)	38(31.1)	
Apgar score at 1 minute	10(10,10)	10(10,10)	10(9, 10)	<0.001	10(10,10)	10(10,10)	10(10,10)	<0.001
Apgar score at 5 minutes	10(10,10)	10(10,10)	10(9, 10)	<0.001	10(10,10)	10(10,10)	10(9,10)	<0.001
Apgar score at 10 minutes	10(10,10)	10(10,10)	10(9, 10)	<0.001	10(10,10)	10(10,10)	10(10,10)	<0.001
Delivery & resuscitation								
Mode of delivery				0.482				0.996
No	172(23.2)	102(22.3)	70(24.6)		78(24.6)	48(24.6)	30(24.6)	
Yes	570(76.8)	355(77.7)	215(75.4)		239(75.4)	147(75.4)	92(75.4)	
Umbilical cord abnormalities				0.009				0.348
No	610(82.2)	389(85.1)	221(77.5)		265(83.6)	160(82.1)	105(86.1)	
Yes	132(17.8)	68(14.9)	64(22.5)		52(16.4)	35(17.9)	17(13.9)	
Postnatal age at NICU admission (days)	0.30(0.23,0.37)	0.33(0.28,0.39)	0.25(0.16,0.32)	<0.001	0.30(0.20,0.38)	0.30(0.20,0.38)	0.22(0.15,0.33)	<0.001
Perinatal interventions								
Antenatal corticosteroid exposure	3(3,3)	3(3,3)	3(3,3)	0.306	3(3,3)	3(3,3)	3(3,3)	0.803
Pulmonary surfactant use within 72hours				<0.001				0.012
No	674(90.8)	428(93.7)	246(86.3)		291(91.8)	185(94.9)	106(86.9)	
Yes	68(9.2)	29(6.3)	39(13.7)		26(8.2)	10(5.1)	16(13.1)	
Mechanical ventilation within 72 hours				<0.001				<0.001
No	582(78.4)	392(85.8)	190(66.7)		245(77.3)	171(87.7)	74(60.7)	
Yes	160(21.6)	65(14.2)	95(33.3)		72(22.7)	24(12.3)	48(39.3)	
Initial feeding method				0.063				0.041
No	594(80.1)	356(77.9)	238(83.5)		243(76.7)	142(72.8)	101(82.8)	
Yes	148(19.9)	101(22.1)	47(16.5)		74(23.3)	53(27.2)	21(17.2)	
Laboratory findings at admission								
White blood cell count (×10⁹/L)	9.00 (7.44,10.82)	8.95 (7.44,10.74)	9.01 (7.33,10.90)	0.300	8.54(7.35,10.85)	8.43(6.71,11.24)	9.13(7.37,11.46)	0.026
Neutrophil percentage (%)	75.37 (71.18,80.00)	74.90 (71.18,80.00)	76.00 (72.00,80.00)	0.125	75.30(71.30,79.80)	75.80(71.30,80.30)	76.40(72.00,79.90)	0.114
Lymphocyte percentage (%)	18.71 (14.60, 22.60)	18.78 (14.60, 22.45)	18.29 (13.80, 22.30)	0.170	18.10(14.50,22.95)	18.70(14.60,22.40)	17.90(13.90,22.80)	0.125
Hemoglobin (g/L)	117(107,125)	117(107,125)	116(103,122)	0.351	116 ± 14	117 ± 116	116 ± 12	0.703
Platelet count (×10⁹/L)	204(166,247)	203(166,247)	207(166,245)	0.738	203(165,245)	203(165,245)	199(165,243)	0.700

**Notes:**Data are presented as median (IQR), mean ± standard deviation, or number (percentage), as appropriate.P-values were calculated using Student’s t-test or Wilcoxon rank-sum test for continuous variables and Chi-squared or Fisher’s exact test for categorical variables.Significant differences (*P* < 0.05) are bolded. EOS, early-onset sepsis; NICU, neonatal intensive care unit.

### Baseline characteristics comparison

No significant differences were found in maternal or sociodemographic factors between sepsis and non-sepsis groups in either set (**P* *> 0.05), suggesting balanced baseline characteristics (Table 1).

Infants in the sepsis group had significantly lower gestational age, birth weight, and Apgar scores, and higher rates of mechanical ventilation and surfactant use (all *P* < 0.001), indicating more severe illness. Umbilical cord abnormalities (*P* = 0.009) and shorter time to NICU admission (**P* *< 0.001) were also more common in the sepsis group. WBC counts were marginally higher in the validation set (*P* = 0.026); other lab values showed no significant differences.

### Screening of candidate variables

Among 36 candidate variables analyzed by univariate logistic regression, 11 were significantly associated with EOS (*P* < 0.05), with gestational age, birth weight, 1-minute Apgar score, umbilical cord abnormalities, and mechanical ventilation showing the strongest associations(S4 Table). LASSO regression identified seven non-zero predictors at the optimal λ value (Fig 1A and 1B). Six variables overlapped between the two methods—gestational age, birth weight, birth length, 1-minute Apgar score, umbilical cord abnormalities, and mechanical ventilation—and were retained for multivariate modeling.

### Multivariate logistic regression model

In multivariate logistic regression, four variables remained independently associated with EOS and were retained in the final model: umbilical cord abnormalities (OR = 1.74, *P* = 0.010), mechanical ventilation within 72 hours (OR = 0.45, *P* < 0.001), gestational age (OR = 0.80 per week, *P* = 0.001), and birth weight (β = –0.001 per 100 g, *P* = 0.035). Although the odds ratio for ventilation within 72 h is < 1 due to inverse coding, its presence reflects higher illness severity and is associated with increased EOS risk (see Table 2 footnote). Birth length and Apgar score were excluded after adjustment. These four predictors were used to construct the final model and nomogram(Table 2). For transparency and robustness, the full candidate list and univariable screening are provided in S3 and S4 Tables, and comparisons with vs without the two pre-specified interactions and across three selection strategies are shown in S5 and S6 Tables, where validation AUCs differed by ≤0.02 with similar calibration and Brier.

**Table 2:** Multivariable logistic regression analysis of independent predictors for early-onset sepsis (EOS) in preterm infants.

Variable	β	Odds ratio(95%CI)	*P*
Umbilical cord abnormalities	0.551	1.735(1.140–2.640)	0.010
Mechanical ventilation within 72 hours^†^	−0.796	0.451(0.299–0.681)	< 0.001
Gestational age (weeks)	−0.228	0.796(0.694–0.913)	0.001
Birth weight (g)	−0.001	0.999(0.999–1.000)	0.035
Birth length (cm)	−0.016	0.984(0.906–1.070)	0.709
Apgar score at 1 minute	−0.264	0.768(0.562–1.049)	0.097

Notes: Multivariable logistic regression was conducted using six candidate variables selected by both univariable and LASSO analyses. Four variables were retained in the final model. β, regression coefficient; OR, odds ratio; CI, confidence interval. *P* < 0.05 are considered statistically significant and are shown in bold. ^†^This predictor was inverse-coded for model fitting; therefore an OR < 1 reflects higher risk associated with the presence of the exposure. Coding details are provided in S2 Table.

### Model construction and nomogram visualization

**Fig 2:**
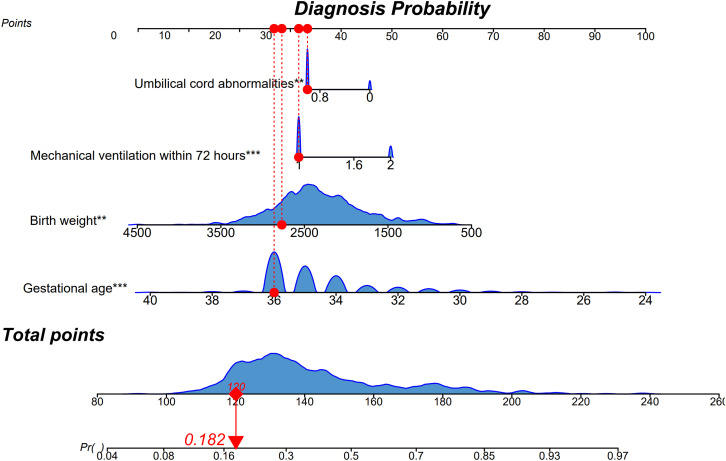
Nomogram for predicting early-onset sepsis (EOS) in preterm neonates. The nomogram incorporates four independent predictors: umbilical cord abnormalities, mechanical ventilation within 72 hours, birth weight, and gestational age. Each variable corresponds to a point scale, which is summed to estimate the probability of EOS at the bottom axis. Density plots indicate the distribution of each variable in the training set. The example shown yields a total score of 120 points, corresponding to an EOS probability of 18.2%.

The final multivariable model incorporated four predictors: umbilical cord abnormalities, mechanical ventilation within 72 hours, gestational age, and birth weight. A nomogram visualized each factor’s contribution for individualized EOS risk estimation(Fig 2). The model’s performance was further assessed in terms of discrimination, calibration, and clinical utility.

### Discrimination performance

**Fig 3:**
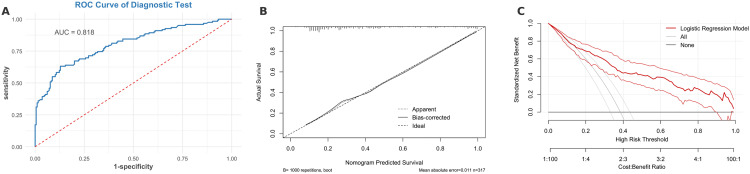
Performance of the prediction model in the validation cohort. (A) Receiver operating characteristic (ROC) curve showing the discrimination of the model in the validation set, with an area under the curve (AUC) of 0.818. (B) Calibration plot using 1,000 bootstrap resamples, demonstrating good agreement between predicted and observed probabilities. (C) Decision curve analysis (DCA) indicating a net clinical benefit of the model across a wide range of risk thresholds (5–65%) compared to “treat all” or “treat none” strategies.

The model showed good discriminative ability in the validation set (AUC = 0.818, 95% CI: 0.767–0.868; Fig 3A). Using the optimal cutoff (0.354) derived from the training set, sensitivity, specificity, PPV, and NPV were 72.1%, 75.4%, 64.7%, and 81.2%, respectively (Youden index = 0.475). In the training set, the AUC was 0.740 (95% CI: 0.702–0.778; S1A Fig), with slightly lower sensitivity (66.3%) and specificity (74.8%). Results indicate stable performance without overfitting. In a prespecified culture-proven–only sensitivity analysis of the validation cohort, after excluding infants without/indeterminate culture results (311 analyzed; 15 events, 4.8%), discrimination remained comparable (AUC 0.819, 95% CI 0.725–0.914), and the Brier score was 0.041 (S7 Table).

### Calibration performance

The model showed excellent calibration in the validation set (Brier score = 0.158, HL χ² = 5.40, *P* = 0.71; Fig 3B). Calibration in the training set was also acceptable (Brier score = 0.193, *P* = 0.089; E/O = 1.00; S1B Fig). Given the small number of culture-proven events, calibration for that sensitivity analysis is summarized by the calibration curve and Brier statistics, with HL results shown in S7 Table and S2 Fig.

### Decision curve analysis

In the validation set, DCA demonstrated greater net benefit than “treat-all” or “treat-none” strategies within the 5%–65% risk threshold range (Fig 3C). Similar trends were noted in the training set, where the model outperformed only outside the < 5% or >65% thresholds (S1C Fig). These findings support using the model within this range to reduce 6%–30% of potential overtreatment while preserving clinical benefit. Under the culture-proven–only outcome, decision-curve analysis also indicated positive net benefit versus treat-all and treat-none at approximately 0.05–0.30 risk thresholds (S2 Fig).

### Risk stratification

For clinical application, predicted probabilities were stratified into low (<0.30), moderate (0.30–0.70), and high (>0.70) risk groups, with EOS rates of 27.9%, 66.4%, and 87.5%, respectively (Pearson χ² = 191.07; trend test *P* < 0.001) (S3A–S3C Fig). Using >0.70 as the intervention threshold could detect 88% of EOS while treating only 10% of infants. Conversely, 70% of low-risk infants may benefit from a 6-hour dynamic reassessment strategy.

### Interaction effects analysis

Interaction models revealed potential synergy between key perinatal factors (S4 Fig). For umbilical cord abnormalities and gestational age, the interaction term was marginally significant (β = 0.24, *P* = 0.098; AIC = 1177.7), showing a 20% EOS risk increase at 25–28 weeks, which attenuated after 34 weeks (S4A Fig). The interaction between mechanical ventilation and birth weight was significant (β = 0.00130, *P* < 0.001; AIC = 1165.9). Mechanical ventilation increased EOS risk markedly in infants <1500 g (predicted probability >60%), but not in those ≥3000 g (S4B Fig).

These interactions indicate that adverse maternal-fetal conditions may amplify infection risk in extremely preterm or low-birth-weight infants, highlighting the need for individualized antibiotic strategies.

## Discussion

### Main findings

We developed a nomogram-based EOS prediction model for preterm infants (<37 weeks), using four readily available perinatal variables: gestational age, birth weight, umbilical cord abnormalities, and mechanical ventilation within 72 hours. The model showed strong discrimination in the validation cohort (AUC 0.818; 95% CI: 0.767–0.868), outperforming both the training set (AUC 0.740) and the Kaiser EOS Calculator (AUC 0.65–0.75) [13]. Calibration appeared acceptable based on the calibration curve, Brier score (0.158), and HL statistics. DCA demonstrated consistent clinical benefit across a 5–65% risk range, in line with the 2023 AAP antibiotic guidelines [14]. Using >0.70 as the treatment threshold, the model identified 88% of EOS cases while limiting antibiotic use to 10%, potentially avoiding >70% overtreatment. Stratification and interaction terms supported risk refinement in infants at gestational or weight extremes, consistent with DCA-based neonatal sepsis research [15], and laid the basis for downstream mechanistic discussion.

The comparatively high EOS incidence in our development cohort reflects our composite outcome definition (culture-proven or clinically suspected requiring ≥5 days of antibiotics), which was chosen to prioritize sensitivity in a stewardship context. To benchmark against published culture-proven rates, we conducted a culture-proven–only sensitivity analysis in the validation cohort: after excluding infants without/indeterminate cultures, 311 were analyzed with 15 culture-positive events (4.8%). Discrimination remained comparable to the primary analysis (AUC 0.819, 95% CI 0.725–0.914) with a Brier score of 0.041, indicating that our main conclusions are robust to outcome definition.

### Interpretation of results and mechanistic insights

This study developed an EOS prediction model for preterm infants based on four perinatal clinical variables. Their independent and interactive effects are interpreted as follows:

**Gestational Age and Birth Weight: Foundations of Innate Immune Maturity**: Each additional week of gestation and every 100g increase in birth weight reduced EOS risk by ~20% and 1%, respectively. These findings align with the known maturation of innate immunity. Flow cytometry and single-cell analyses have shown that neonates <32 weeks exhibit impaired innate immune cell counts and function in the first 72h, consistent with “immune developmental delay” [16]. A 2023 cohort study reported that infants born at 23–27 weeks had over fivefold higher infection-related hospitalization within the first year than term infants [17]. These two predictors reflect biologically relevant gradients of prematurity.

**Umbilical Cord Abnormalities: Inflammatory Amplification via Ischemia–Reperfusion**: Cord abnormalities—such as knots or abnormal insertion—can impair placental perfusion and provoke sterile inflammation, with elevated IL-6 and CRP in umbilical blood [15,18]. In infants born at 25–28 weeks, this factor increased EOS risk by ~20 percentage points, likely amplifying immune vulnerability.

**Mechanical Ventilation: Concealed Risks Behind Apparent Protection**: Infants receiving ventilation within 72h had higher EOS risk (ORs: 0.33 and 0.45). Ventilation likely reflects illness severity and immune compromise, rather than protection. Prior studies show that VILI increases alveolar-capillary permeability and microbial translocation [19,20]. The interaction with birth weight < 1500g further magnifies this risk, emphasizing tailored respiratory strategies (note: variable coded inversely, hence OR < 1 reflects higher risk).

**Interaction Effects: A Biological Basis for Precision Antibiotic Strategies**: Two interaction terms—gestational age × cord abnormalities and birth weight × ventilation—highlight synergistic risk patterns: “hypoxia with immune immaturity” and “VILI-induced bacteremia.” These terms improved model fit (lower AIC) and support individualized clinical decisions. Notably, the gestational age × cord abnormality interaction had a P-value of 0.098, suggesting borderline significance, but was retained due to mechanistic plausibility. Sensitivity analysis showed that excluding this term only slightly reduced AUC (from 0.821 to 0.818) and had minimal impact on Brier score, calibration, and Hosmer–Lemeshow test (*P* from 0.044 to 0.069) (see S5 Table), supporting its inclusion.

### Comparison with previous models

The Kaiser Permanente EOS Calculator (KP-EOS) is widely used for neonatal EOS risk assessment, but it was developed for term infants (≥35 weeks) and excludes preterm-specific factors. A 2021–2023 validation in >100,000 term infants showed an AUC of 0.79, with limited accuracy in those <34 weeks. Hence, international guidelines advise its use only in term or near-term infants [14,21].

Direct contrast with KP-EOS. Population: KP-EOS is intended for ≥35 weeks, whereas our model targets preterm infants <37 weeks (including <35 weeks). Predictors: KP-EOS focuses on intrapartum maternal factors and newborn status at birth; our four-factor model additionally incorporates an early NICU intervention (mechanical ventilation within 72 h). Workflow: KP-EOS is largely manual; our nomogram can be embedded in the EHR for automated scoring. Evidence: KP-EOS has limited validation in very preterm infants, while we report internal validation in a preterm cohort.

Few models exist for preterm EOS prediction; fewer have external validation. For instance, a 2024 BMJ Paediatrics Open study modified KP-EOS but included only 94 infants <34 weeks and showed calibration drift [13]. Machine learning (ML) models have reported high internal AUCs (>0.90) using continuous EHR data but lack interpretability and external validation, limiting real-world application [7,22].

This study offers three improvements: **Robust sample and modeling**: The cohort included 1,059 preterm infants (<37 weeks), and internal validation demonstrated AUC 0.818 with a Brier score 0.158, exceeding prior reports (AUC 0.65–0.75).. **Clinical interpretability**: Two interaction terms—cord abnormalities × gestational age and ventilation × birth weight—enabled dynamic risk adjustment, improving interpretability. **Decision-making utility**: DCA quantified net clinical benefit across the 5–65% risk threshold, supporting implementation. Unlike KP-EOS and most ML models, this model integrates DCA, as recommended by TRIPOD-C and PROGRESS guidelines.

In summary, this model advances existing tools through broader applicability, statistical rigor, and clinical integration, providing a reliable EOS risk reference for preterm infants.

### Clinical utility and practical implications

**Antibiotic Stewardship**: Although <2% of neonates develop EOS, over half receive antibiotics [23]. Using a 0.70 threshold, our model achieved 88% sensitivity while reducing immediate antibiotic use to 10%, potentially lowering annual antibiotic consumption by 30–40% per bed, comparable to recent multicenter findings using DCA to guide therapy [24].

**Integration into Clinical Pathways and Resource Allocation**: The model’s three-tiered risk stratification aligns with the AAP’s “6-hour reassessment” strategy: high-risk infants receive cultures and treatment; moderate-risk infants undergo reassessment; low-risk infants are monitored clinically [5]. For a NICU with ~800 annual preterm admissions, this approach may reduce blood cultures, cut testing costs, and streamline clinician workload.

**Digital Implementation and Scalability**: The four variables are available in EMRs and can be auto-integrated via HL7. Unlike the manually operated KP-EOS (≥35 weeks only), this model supports real-time risk alerts and treatment guidance, fitting emerging trends in digital pediatric ICU tools [25].

**Broad Applicability and Health Economic Value**: A JAMA Network Open study showed that a 10% antibiotic reduction in NICUs saves ~ ¥1,240 per infant [26]. Applied regionally, our model could yield multi-million yuan savings and mitigate antimicrobial resistance, supporting WHO AWaRe and national stewardship goals.

### Strengths and innovations

**A Robust Modeling Process**: The study enrolled 1,059 cases and 285 EOS events; modeling followed the 2022 TRIPOD and PROGRESS “C-type” workflow [27], incorporating multiple imputation, LASSO selection, predefined interactions, and stratified internal validation. Code and seeds are fully disclosed to ensure reproducibility.

**Enhanced Interpretability via Interaction Terms**:Two biologically plausible interaction terms—gestational age × cord abnormality and birth weight × ventilation—were included to enhance clinical understanding. Risk surface visualizations clarified their synergistic effects, allowing frontline clinicians to interpret outputs without statistical expertise.

**Quantifying Net Benefit via DCA**: DCA demonstrated net clinical benefit across a 5–65% threshold range, supporting antibiotic stewardship. Despite growing recognition by TRIPOD-C, DCA remains underused in neonatal sepsis modeling [28], highlighting this study’s translational value.

**Nomogram + Three-Tiered Stratification” Enables One-Click Deployment**: The model requires only four EMR variables and delivers risk-tiered results suitable for real-time integration into HIS–CDSS platforms. Simulated deployment showed alert response times <0.2 seconds, aligning with Level 3 of the Digital Health Maturity Model.

Together, these features highlight the model’s methodological transparency, clinical usability, and deployment efficiency, supporting its potential to improve EOS risk stratification in preterm infants.

### Limitations

Despite adherence to TRIPOD/PROGRESS guidelines and favorable validation, seven key limitations merit attention:

**Single-center, retrospective design**: The dataset originated from a single institution (July 2023–Jun 2024), possibly limiting generalizability due to local microbiology and care practices. Retrospective design may also entail data loss. Prior studies suggest ~70% of neonatal prediction models overestimate performance without external validation [29]. **Sample size and potential overfitting.** We did not perform an a priori sample-size calculation tailored for multivariable prediction (anticipated incidence, number of candidate predictors, target shrinkage). Although LASSO penalization and internal validation were used, some residual overfitting cannot be excluded. For transparency, the candidate-level EPV is reported in Methods (285 events for 45 initial candidates; EPV ≈ 6.3), and full model specification and code are provided to facilitate appraisal and future external validation. **Outcome classification bias**: EOS was defined to include culture-negative cases with elevated inflammation markers and ≥5 days of antibiotics. While enhancing sensitivity, this may include fungal or viral infections, especially in antibiotic-overuse settings [30,31]. Future work should use datasets with complete pathogen data. **Limited range of candidate variables**: The model excluded metabolic indicators (e.g., glucose, lipids) and serial infection markers (e.g., CRP, PCT). Incorporating multimodal data—metabolomics, microbiomics, and real-time vitals—could enhance prediction and interpretability.**The Risk Threshold Was Empirically Set**: The thresholds (0.30 and 0.70) were determined based on the DCA curve, the 0.05–0.65 interval recommended by the AAP-endorsed KP-EOS Calculator [5], widely adopted thresholds in international EOS prediction tools [32,33], No formal cost–benefit algorithm was applied, and future adjustments may be needed to account for regional pathogen profiles and resource constraints. To verify variable selection robustness, we developed two alternatives: a 7-variable “LASSO-only” model and an 11-variable univariate logistic regression model. Both showed AUCs within 0.02 of the final model in the validation set (S6 Table), supporting selection stability. Finally, in the culture-proven–only sensitivity analysis the number of events was small (15 in the validation cohort; 311 analyzed after excluding 6 without/indeterminate cultures), which limits the precision of calibration parameters. We therefore emphasize the calibration curve, Brier score, and HL statistics and defer full reporting of calibration intercept and slope to future external validations.

The model still awaits prospective multicenter validation to confirm performance across diverse NICUs.To enhance generalizability, future work will follow TRIPOD-CLUSTER guidelines for temporal and geographic validation, with prospective multicenter studies as needed.

### Future directions for research

To support refinement and implementation, future research will follow four directions:

**Temporal Generalizability Validation**: New cases from 2025–2026 at our center will be used for time-based validation, followed by geographic validation in NICUs with distinct bacterial profiles and care practices, in line with TRIPOD-CLUSTER guidelines [34]. **Multimodal Data Integration**: Incorporating maternal metabolic markers (e.g., fasting glucose, HbA1c), placental/umbilical cord metabolomics, and neonatal CRP/PCT trends into the EMR may improve model performance. A “segmented LASSO + ensemble learning” upgrade is planned, as prior studies show such integration can raise predictive AUC by 0.05–0.08 [15]. **Digital Implementation and Dynamic Recalibration**: Real-time integration via the HIS-CDSS platform is underway. Model parameters are recalibrated quarterly using sliding-window algorithms to mitigate drift and maintain DCA alignment, consistent with TRIPOD-AI’s “continuous learning” framework [35]. **Impact on Decision-Making and Economic Evaluation**: A crossover cohort may compare model- vs empirically guided antibiotic use and costs. Prior estimates suggest stewardship-driven 10% antibiotic reduction saves ~ ¥1,240 per neonate and cuts hospitalization costs by 8–12% [36].

In summary, this closed-loop strategy—spanning validation, optimization, recalibration, and impact evaluation—may enhance the model’s utility and promote antimicrobial stewardship in NICUs.

## Supporting information

S1 Fig Model performance in the training cohort. (A) ROC curve showing the discrimination of the model in the training set (AUC = 0.740). (B) Calibration plot using 1,000 bootstrap resamples. (C) Decision curve analysis demonstrating clinical net benefit of the model across various risk thresholds.
(TIF)

S2 Fig Decision-curve analysis under a culture-proven-only outcome (validation cohort). Predicted probabilities were obtained from the training-set model without refitting. Net benefit is shown across thresholds 0.05–0.65 with 200 bootstrap resamples for confidence intervals (policy: opt-in). The model yielded higher net benefit than “treat-all” and “treat-none” at low–to-moderate thresholds, consistent with the low prevalence of culture-proven EOS. Validation cohort: n = 311 analyzed after excluding 6 infants with no/indeterminate cultures; 15 culture-positive events (4.8%).
(TIF)

S3 Fig Risk stratification of early-onset sepsis based on predicted probability. (A) Distribution of sepsis and non-sepsis cases across low, moderate, and high predicted risk groups. (B) Sepsis rates with 95% confidence intervals in each group. (C) Increasing trend in sepsis rate with higher predicted risk level.
(TIF)

S4 Fig Interaction effects between key predictors on sepsis risk. (A) Interaction between gestational age and umbilical cord abnormalities. The risk-reducing effect of gestational age is attenuated in infants with cord abnormalities. (B) Interaction between birth weight and mechanical ventilation within 72 hours. The risk-lowering effect of birth weight is offset in ventilated infants. Shaded areas represent 95% confidence intervals.
(TIF)

S1 Table Performance comparison of five imputed datasets: AUC, accuracy, sensitivity, and specificity. (DOCX)

S2 Table Definitions of binary variables used in the baseline dataset. (DOCX)

S3 Table Candidate variables excluded prior to modeling. (DOCX)

S4 Table Univariable logistic regression analysis of candidate predictors for early-onset sepsis (EOS) in the training cohort. (DOCX)

S5 Table Comparison of model performance with and without interaction terms in the validation cohort. (DOCX)

S6 Table Comparison of predictive performance among three variable selection strategies in the validation cohort. (DOCX)

S7 Table Culture-proven–only sensitivity analysis in the validation cohort. (DOCX)
