# Supplementary figures and images for: Four-factor nomogram for early-onset sepsis in preterm neonates: Development and internal validation of a stewardship tool
Source: PLoS One. 2025 Oct 9;20(10):e0334342. doi: 10.1371/journal.pone.0334342 (PMC12510551; doi:10.1371/journal.pone.0334342)

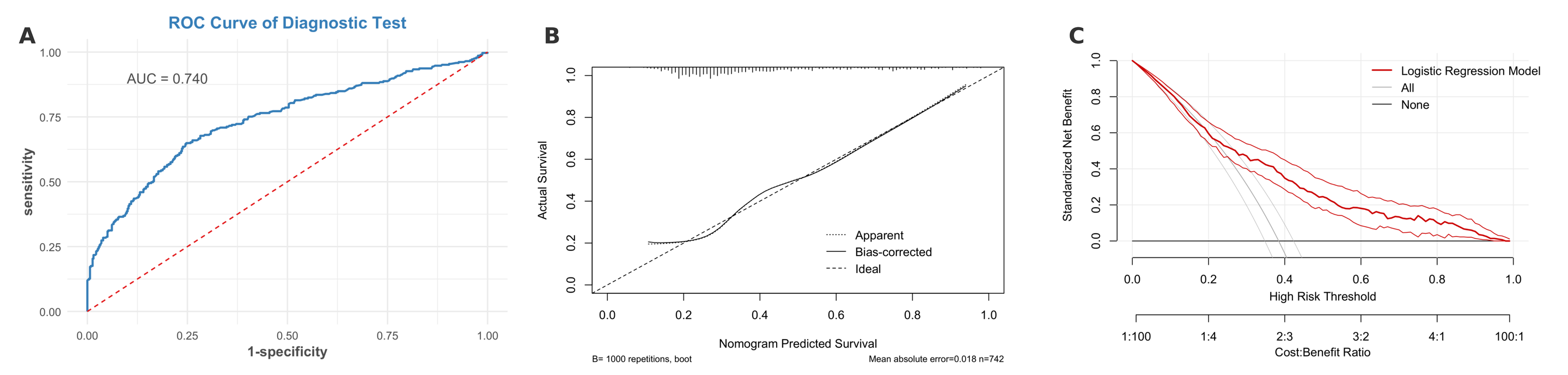

Supplement: S1 Fig — (A) ROC curve showing the discrimination of the model in the training set (AUC = 0.740). (B) Calibration plot using 1,000 bootstrap resamples. (C) Decision curve analysis demonstrating clinical net benefit of the model across various risk thresholds. (TIF) [file pone.0334342.s001.tif]

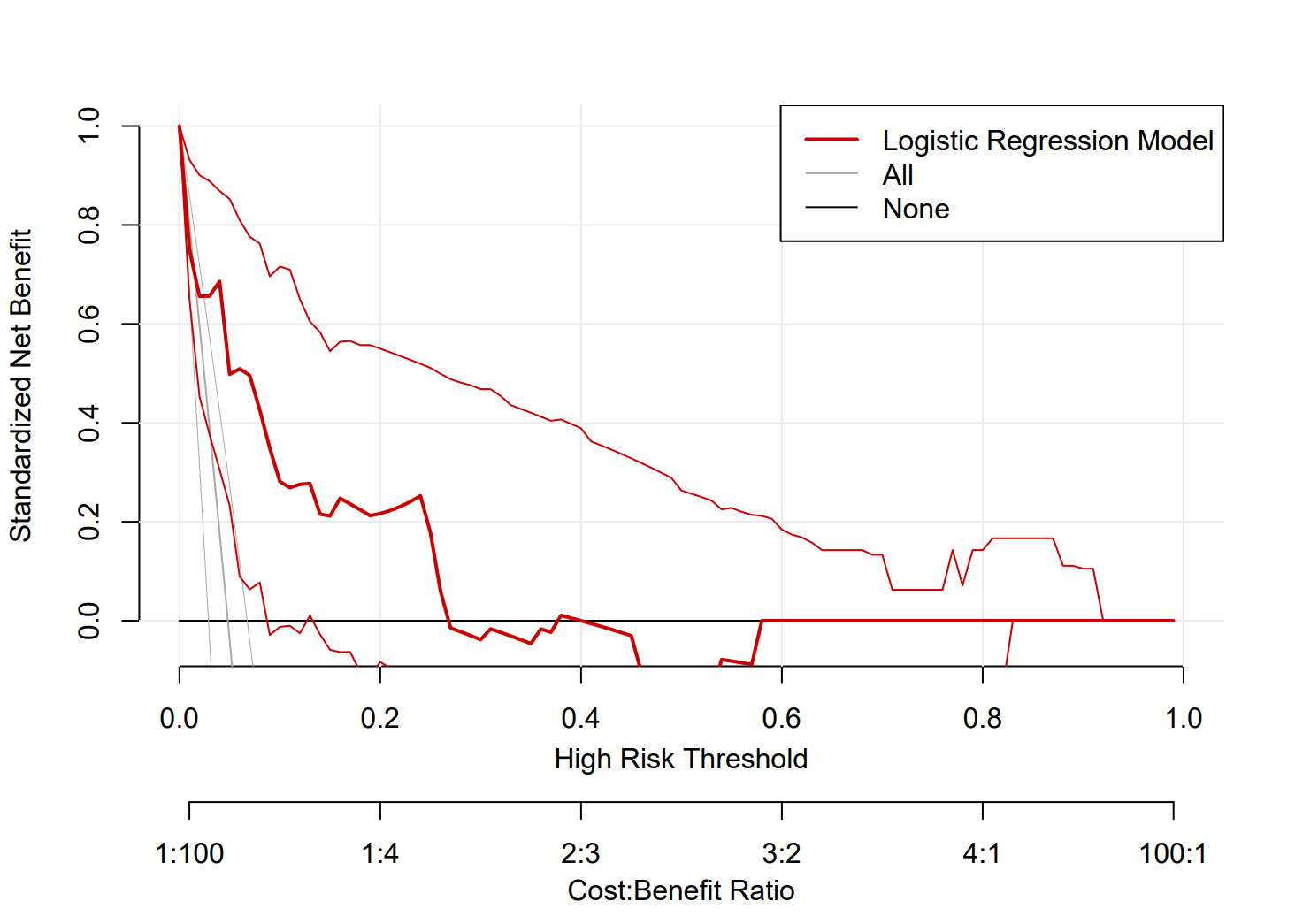

Supplement: S2 Fig — Predicted probabilities were obtained from the training-set model without refitting. Net benefit is shown across thresholds 0.05–0.65 with 200 bootstrap resamples for confidence intervals (policy: opt-in). The model yielded higher net benefit than “treat-all” and “treat-none” at low–to-moderate thresholds, consistent with the low prevalence of culture-proven EOS. Validation cohort: n = 311 analyzed after excluding 6 infants with no/indeterminate cultures; 15 culture-positive events (4.8%). (TIF) [file pone.0334342.s002.tif]

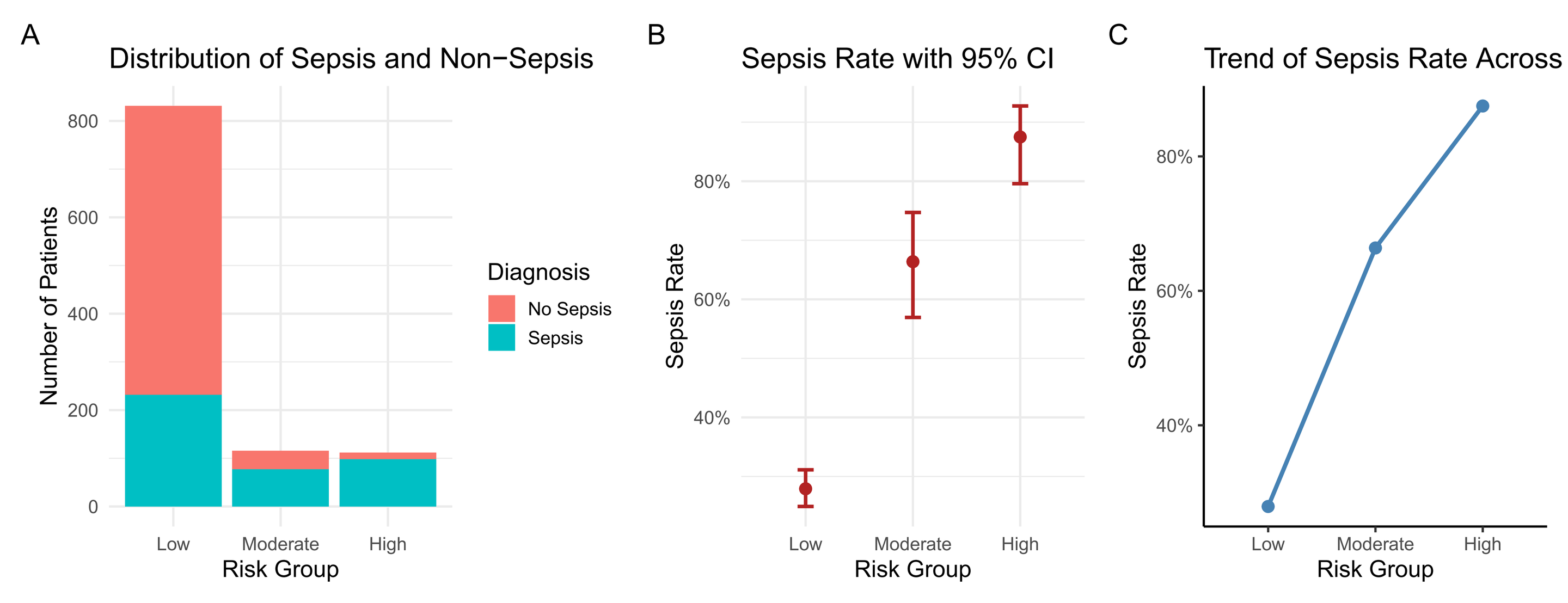

Supplement: S3 Fig — (A) Distribution of sepsis and non-sepsis cases across low, moderate, and high predicted risk groups. (B) Sepsis rates with 95% confidence intervals in each group. (C) Increasing trend in sepsis rate with higher predicted risk level. (TIF) [file pone.0334342.s003.tif]

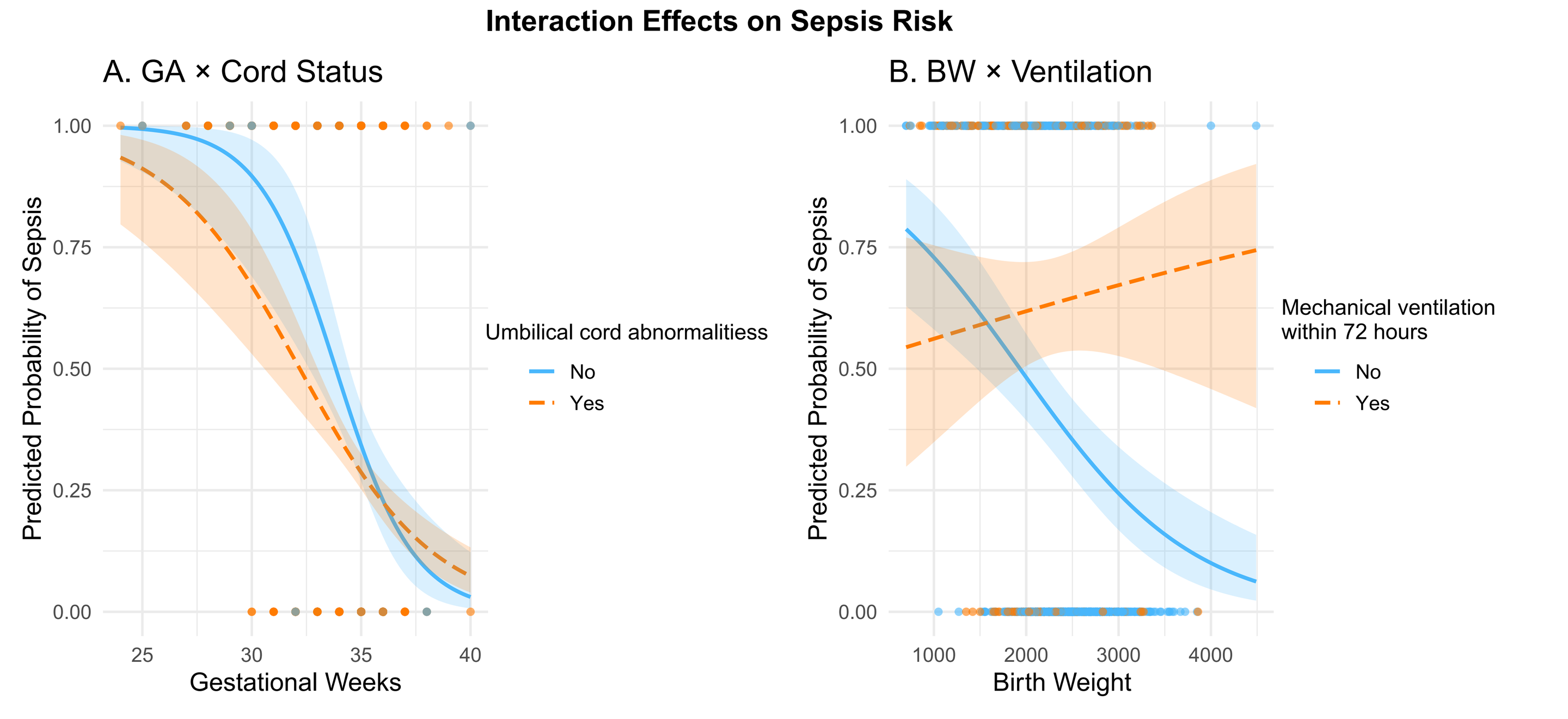

Supplement: S4 Fig — (A) Interaction between gestational age and umbilical cord abnormalities. The risk-reducing effect of gestational age is attenuated in infants with cord abnormalities. (B) Interaction between birth weight and mechanical ventilation within 72 hours. The risk-lowering effect of birth weight is offset in ventilated infants. Shaded areas represent 95% confidence intervals. (TIF) [file pone.0334342.s004.tif]
