# Supplementary material for: Four-factor nomogram for early-onset sepsis in preterm neonates: Development and internal validation of a stewardship tool
Source: PLoS One. 2025 Oct 9;20(10):e0334342. doi: 10.1371/journal.pone.0334342 (PMC12510551; doi:10.1371/journal.pone.0334342)
Supplement: S1 Table — (DOCX) [file pone.0334342.s005.docx]

Supplementary Table 1: Performance Comparison of Five Imputed Datasets: AUC, Accuracy, Sensitivity, and Specificity

| Data Set | AUC | Accuracy | Sensitivity | Specificity |
| --- | --- | --- | --- | --- |
| Data1 | 0.7777 | 0.737 | 0.634 | 0.801 |
| Data2 | 0.7768 | 0.737 | 0.636 | 0.801 |
| Data3 | 0.7766 | 0.736 | 0.636 | 0.798 |
| Data4* | 0.7778 | 0.737 | 0.629 | 0.805 |
| Data5 | 0.7775 | 0.737 | 0.629 | 0.805 |

Supplementary Table 1 presents the model performance (AUC, accuracy, sensitivity, specificity) across five imputed datasets generated from the original data. Given the negligible differences in performance across datasets, **Dataset 4** (denoted with an asterisk) was randomly selected for further analysis.
