# Supplementary material for: Four-factor nomogram for early-onset sepsis in preterm neonates: Development and internal validation of a stewardship tool
Source: PLoS One. 2025 Oct 9;20(10):e0334342. doi: 10.1371/journal.pone.0334342 (PMC12510551; doi:10.1371/journal.pone.0334342)
Supplement: S2 Table — (DOCX) [file pone.0334342.s006.docx]

Supplementary Table 2. Definitions of Binary Variables Used in the Baseline Dataset.

| Variable | Definition of "Yes" | Definition of "No" |
| --- | --- | --- |
| Maternal occupation | Employed (including any form of paid work) | Unemployed (including homemakers and students) |
| Mode of conception | Natural conception | Assisted reproductive technology (e.g., IVF) |
| Abnormal placental pathology | Presence of any pathological placental findings (e.g., chorioamnionitis, infarction, calcification) | No pathological placental findings |
| Singleton pregnancy | Singleton | Multiple gestation |
| Mode of delivery | Vaginal delivery | Cesarean section |
| Umbilical cord abnormalities | Presence of umbilical cord anomalies (e.g., torsion, knots, prolapse) | No cord anomalies |
| Initial feeding method | Administration of oropharyngeal colostrum immunotherapy | No oropharyngeal colostrum administered |

**Note:** Binary variables were coded as "Yes" and "No" according to the definitions above. All definitions were determined based on standardized clinical criteria and chart documentation to ensure consistency across data extraction and analysis. For model fitting, mechanical ventilation within 72 h was inverse-coded (0 = “Yes”, 1 = “No”), so an OR < 1 indicates higher risk associated with ventilation. The clinical definitions of “Yes/No” remain as shown above.
