# Supplementary material for: Four-factor nomogram for early-onset sepsis in preterm neonates: Development and internal validation of a stewardship tool
Source: PLoS One. 2025 Oct 9;20(10):e0334342. doi: 10.1371/journal.pone.0334342 (PMC12510551; doi:10.1371/journal.pone.0334342)
Supplement: S3 Table — (DOCX) [file pone.0334342.s007.docx]

Supplementary Table 3. Candidate Variables Excluded Prior to Modeling

A total of 45 candidate variables were initially extracted, encompassing maternal demographics, obstetric history, delivery details, perinatal interventions, and neonatal laboratory findings. Of these, 9 variables were excluded before univariate analysis for one or more of the following reasons:
(i) >5% missingness;
(ii) lack of early availability in clinical workflows;
(iii) reverse temporal association with outcome (i.e., post-EOS); or
(iv) poor clinical relevance or ambiguous definition.

These excluded variables are summarized below:

| **Variable name** | **Reason for exclusion** |
| --- | --- |
| Neonatal antibiotic exposure (after birth) | Post-outcome variable; could bias causality |
| Hospital expense | Related to outcome; not early available |
| Hospital stay (hospital day) | Post-EOS; circular causality |
| CRP level | Not routinely measured within 72h; high missingness |
| Time to initiation of feeding | Unrelated to EOS pathophysiology |
| Maternal education level | Not collected consistently |
| Maternal BMI | Not documented in all cases |
| Chorioamnionitis | Diagnosis subjective; high variability |
| Neonatal infection (discharge diagnosis) | Part of outcome; reverse causality |
