# Supplementary material for: Four-factor nomogram for early-onset sepsis in preterm neonates: Development and internal validation of a stewardship tool
Source: PLoS One. 2025 Oct 9;20(10):e0334342. doi: 10.1371/journal.pone.0334342 (PMC12510551; doi:10.1371/journal.pone.0334342)
Supplement: S4 Table — (DOCX) [file pone.0334342.s008.docx]

Supplementary Table 4. Univariable logistic regression analysis of candidate predictors for early-onset sepsis (EOS) in the training cohort

|  |  |  |  |
| --- | --- | --- | --- |
| Variable | β | Odds ratio(95%CI) | *P* |
| Maternal demographics |  |  |  |
| Maternal age (years) | -0.006 | 0.994(0.959–1.030) | 0.731 |
| Maternal occupation | 0.030 | 1.031(0.764–1.389) | 0.844 |
| Maternal smoking | 0.039 | 1.040(0.247–4.385) | 0.958 |
| Gravidity(n) | -0.031 | 0.969(0.799–1.176) | 0.751 |
| Parity(n) | -0.060 | 0.942(0.788–1.125) | 0.508 |
| Mode of conception | 0.245 | 1.278(0.842–1.940) | 0.249 |
| Maternal complications |  |  |  |
| Hypertensive disorders in pregnancy | –0.205 | 0.815(0.587–1.131) | 0.221 |
| Gestational diabetes mellitus | 0.093 | 1.097(0.785–1.534) | 0.586 |
| Intrapartum fever | -0.713 | 0.490(0.191–1.257) | 0.138 |
| Premature rupture of membranes | -0.010 | 0.990(0.715–1.371) | 0.954 |
| Placenta previa | -0.192 | 0.825(0.385–1.771) | 0.622 |
| Placental abruption | -0.140 | 0.869(0.381–1.984) | 0.739 |
| Abnormal placental pathology | -0.143 | 0.867(0.590–1.273) | 0.466 |
| Perinatal characteristics |  |  |  |
| Singleton pregnancy | 0.301 | 1.351(0.978–1.868) | 0.068 |
| Neonatal sex | 0.282 | 1.325(0.983–1.787) | 0.065 |
| Gestational age (weeks) | -0.457 | 0.633(0.573–0.699) | <0.001 |
| Additional gestational days | -0.052 | 0.949(0.881–1.023) | 0.172 |
| Birth weight (g) | -0.002 | 0.998(0.998–0.999) | <0.001 |
| Birth length (cm) | -0.207 | 0.813(0.773–0.855) | <0.001 |
| Head circumference (cm) | -0.356 | 0.701(0.637–0.771) | <0.001 |
| Birth asphyxia | -0.071 | 0.932(0.662–1.311) | 0.684 |
| Apgar score at 1 minute | -0.965 | 0.381(0.294–0.493) | <0.001 |
| Apgar score at 5 minutes | -1.391 | 0.249(0.165–0.375) | <0.001 |
| Apgar score at 10 minutes | -1.360 | 0.257(0.170–0.387) | <0.001 |
| Delivery & resuscitation |  |  |  |
| Mode of delivery | 0.125 | 1.133(0.800–1.605) | 0.482 |
| Umbilical cord abnormalities | 0.505 | 1.657(1.134–2.420) | 0.009 |
| Postnatal age at NICU admission (days) | -4.187 | 0.015(0.004–0.058) | < 0.001 |
| Perinatal interventions |  |  |  |
| Antenatal corticosteroid exposure | -0.253 | 0.777(0.515–1.172) | 0.228 |
| Pulmonary surfactant use within 72hours | -0.850 | 0.427(0.258–0.709) | < 0.001 |
| Mechanical ventilation within 72 hours | -1.104 | 0.332(0.231–0.475) | < 0.001 |
| Initial feeding method | 0.362 | 1.437(0.980–2.107) | 0.064 |
| Laboratory findings at admission |  |  |  |
| White blood cell count (×10⁹/L) | 0.045 | 1.046(0.995–1.099) | 0.075 |
| Neutrophil percentage (%) | 0.017 | 1.018(0.997–1.038) | 0.087 |
| Lymphocyte percentage (%) | -0.016 | 0.984(0.961–1.007) | 0.162 |
| Hemoglobin (g/L) | -0.007 | 0.993(0.983–1.003) | 0.171 |
| Platelet count (×10⁹/L) | <0.001 | 1.000(0.998–1.003) | 0.857 |

**Notes:**Univariable logistic regression was performed to assess the association between each candidate variable and the occurrence of EOS in preterm infants.β, regression coefficient; OR, odds ratio; CI, confidence interval.*P* < 0.05 are considered statistically significant and shown in bold.
