# Supplementary material for: Four-factor nomogram for early-onset sepsis in preterm neonates: Development and internal validation of a stewardship tool
Source: PLoS One. 2025 Oct 9;20(10):e0334342. doi: 10.1371/journal.pone.0334342 (PMC12510551; doi:10.1371/journal.pone.0334342)
Supplement: S5 Table — (DOCX) [file pone.0334342.s009.docx]

Supplementary Table 5. Comparison of model performance with and without interaction terms in the validation cohort.

| **Model Version** | **AUC (95% CI)** | **Intercept** | **Slope** | **Brier Score** | **HL Test P-value** |
| --- | --- | --- | --- | --- | --- |
| Reduced Model | 0.818 (0.767–0.868) | –0.04 | 1.57 | 0.164 | 0.044 |
| Full Model | 0.821 (0.771–0.871) | –0.04 | 1.58 | 0.165 | 0.069 |

**Notes:** The full model includes two predefined interaction terms (gestational age × umbilical cord abnormalities and birth weight × mechanical ventilation within 72 hours). The reduced model excludes these terms.Model performance was assessed in the validation cohort using the area under the ROC curve (AUC), calibration intercept and slope, Brier score, and the Hosmer–Lemeshow (HL) goodness-of-fit test.AUC, area under the curve; HL, Hosmer–Lemeshow.
