# Supplementary material for: Four-factor nomogram for early-onset sepsis in preterm neonates: Development and internal validation of a stewardship tool
Source: PLoS One. 2025 Oct 9;20(10):e0334342. doi: 10.1371/journal.pone.0334342 (PMC12510551; doi:10.1371/journal.pone.0334342)
Supplement: S6 Table — (DOCX) [file pone.0334342.s010.docx]

Supplementary Table 6. Comparison of predictive performance among three variable selection strategies in the validation cohort.

| Model | Number of Predictors | Test‐Set AUC | 95% CI | ΔAUC vs Main Model |
| --- | --- | --- | --- | --- |
| Main Model (Univariable + LASSO) | 4 | 0.802 | 0.751–0.854 | 0.000 |
| LASSO Only (7 variables) | 7 | 0.807 | 0.756–0.858 | +0.005 |
| Univariable Only (*P* < 0.05) | 11 | 0.810 | 0.759–0.861 | +0.008 |

**Notes:**The “Final Model” was constructed based on the intersection of univariable logistic regression (*P* < 0.05) and LASSO-selected predictors (resulting in 4 variables).The “LASSO Only” model included 7 variables with non-zero coefficients at λ<sub>min</sub>.The “Univariable Only” model included all 11 predictors with *P* < 0.05 in univariable analysis.ΔAUC represents the difference in AUC compared to the Final Model; all ΔAUC values were < 0.02, indicating comparable performance.
