# Supplementary material for: Four-factor nomogram for early-onset sepsis in preterm neonates: Development and internal validation of a stewardship tool
Source: PLoS One. 2025 Oct 9;20(10):e0334342. doi: 10.1371/journal.pone.0334342 (PMC12510551; doi:10.1371/journal.pone.0334342)
Supplement: S7 Table — (DOCX) [file pone.0334342.s011.docx]

Supplementary Table 7. Culture-proven–only sensitivity analysis in the validation cohort

| **Metric** | **Value** |
| --- | --- |
| Cohort size (analyzed), n | 311 |
| Excluded for no/indeterminate culture, n | 6 |
| Events (culture-positive), n (%) | 15 (4.8%) |
| AUC (DeLong) | 0.819（0.725-0.914） |
| Brier score | 0.041 |

Notes: Outcome limited to blood-culture–positive EOS within 72 h of birth. Only infants with a documented blood-culture result were analyzed; 6 were excluded for no or indeterminate culture results. Model performance was evaluated on the validation cohort using predicted probabilities from the training-set model. AUC 95% confidence intervals were computed by the DeLong method; the Brier score is reported on a 0–1 scale. Abbreviations: AUC, area under the receiver-operating characteristic curve.
